# FOXA1 Promotes Tumor Progression in Prostate Cancer via the Insulin-Like Growth Factor Binding Protein 3 Pathway

**DOI:** 10.1371/journal.pone.0042456

**Published:** 2012-08-03

**Authors:** Yusuke Imamura, Shinichi Sakamoto, Takumi Endo, Takanobu Utsumi, Miki Fuse, Takahito Suyama, Koji Kawamura, Takashi Imamoto, Kojiro Yano, Katsuhiro Uzawa, Naoki Nihei, Hiroyoshi Suzuki, Atsushi Mizokami, Takeshi Ueda, Naohiko Seki, Hideki Tanzawa, Tomohiko Ichikawa

**Affiliations:** 1 Department of Urology, Chiba University Graduate School of Medicine, Chiba, Japan; 2 Department of Urology, Toho University Medical Center Sakura Hospital, Chiba, Japan; 3 Faculty of Information Science and Technology, Osaka Institute of Technology, Osaka, Japan; 4 Department of Clinical Molecular Biology, Chiba University Graduate School of Medicine, Chiba, Japan; 5 Department of Urology, Kanazawa University Graduate School of Medical Sciences, Ishikawa, Japan; 6 Prostate Center and Division of Urology, Chiba Cancer Center, Chiba, Japan; 7 Department of Functional Genomics, Chiba University Graduate School of Medicine, Chiba, Japan; Florida International University, United States of America

## Abstract

Fork-head box protein A1 (FOXA1) is a “pioneer factor” that is known to bind to the androgen receptor (AR) and regulate the transcription of AR-specific genes. However, the precise role of FOXA1 in prostate cancer (PC) remains unknown. In this study, we report that FOXA1 plays a critical role in PC cell proliferation. The expression of FOXA1 was higher in PC than in normal prostate tissues (P = 0.0002), and, using immunohistochemical analysis, we found that FOXA1 was localized in the nucleus. FOXA1 expression levels were significantly correlated with both PSA and Gleason scores (P = 0.016 and P = 0.031, respectively). Moreover, FOXA1 up-regulation was a significant factor in PSA failure (P = 0.011). Depletion of FOXA1 in a prostate cancer cell line (LNCaP) using small interfering RNA (siRNA) significantly inhibited AR activity, led to cell-growth suppression, and induced G0/G1 arrest. The anti-proliferative effect of FOXA1 siRNA was mediated through insulin-like growth factor binding protein 3 (IGFBP-3). An increase in IGFBP-3, mediated by depletion of FOXA1, inhibited phosphorylation of MAPK and Akt, and increased expression of the cell cycle regulators p21 and p27. We also found that the anti-proliferative effect of FOXA1 depletion was significantly reversed by simultaneous siRNA depletion of IGFBP-3. These findings provide direct physiological and molecular evidence for a role of FOXA1 in controlling cell proliferation through the regulation of IGFBP-3 expression in PC.

## Introduction

Prostate cancer (PC), the most common cancer in men, is a second leading cause of cancer-related death in most of western countries and is becoming more common in Asian countries as well [1]. The hormone androgen and the androgen receptor (AR) are essential for the normal growth, differentiation and maintenance of the prostate gland, and they also play a critical role in the development of PC [2]. Thus, treatment of advanced PC involves blocking the production of androgens or antagonizing AR and its target genes [1]. However, PC relapses after the acquisition of castration resistance. A recent study showed that a low level of intra-tumoral androgen during androgen ablation therapy continues to activate the AR, which leads to further progression of PC into metastasis [3]. Therefore, even at an advanced stage of PC, the regulation of AR activity is important for the treatment of PC.

Based on a previous microarray analysis in which we compared the gene expression of Benign prostate hyperplasia (BPH) and PC, we identified the Fork-head box protein A1 (FOXA1) as a gene whose expression is significantly up-regulated in PC [4]. FOXA1 is a transcription factor that belongs to the human forkhead box gene family, and FOXA1 is involved in endodermal organogenesis, metabolism and homeostasis [5]. FOXA1 directly binds to the AR and regulates the transcription of prostate-specific genes [6]. FOXA1 also serves as a “pioneer factor” for AR at sites of transcriptional regulation [7], [8]. Thus, binding of FOXA1 to modified histone associated with active chromatin facilitates the subsequent recruitment of nuclear receptors and transcriptional activation [7], [8]. Several lines of evidence have linked FOXA1 with prostate development. Epithelial FOXA1 expression has been observed at all stages of development and differentiation of the mouse prostate [6], [9]. The FOXA1-defecient mouse demonstrated loss of prostate morphogenesis and differentiation [9], [10]. FOXA1 also contributes to estrogen signaling in breast cancer, and has been associated with luminal subtype and good prognosis [11], [12]. Increased FOXA1 expression has also been observed in colon, lung, thyroid, esophageal cancer and prostate cancer [13]–[15].

Although FOXA1 associated with prostate development and androgen regulation, the precise mechanism how FOXA1 contribute for PC progression, especially regulation of FOXA1-dependent target genes in PC remain relatively unidentified.

The present study investigated the role of FOXA1 in PC progression. We identified insulin-like growth factor binding protein 3 (IGFBP-3) as a novel target of FOXA1 for the regulation of cell proliferation in PC cells.

## Results

### The Expression of FOXA1 in Prostate Cancer Tissues and its Correlation with Clinical Factors

**Figure 1:**
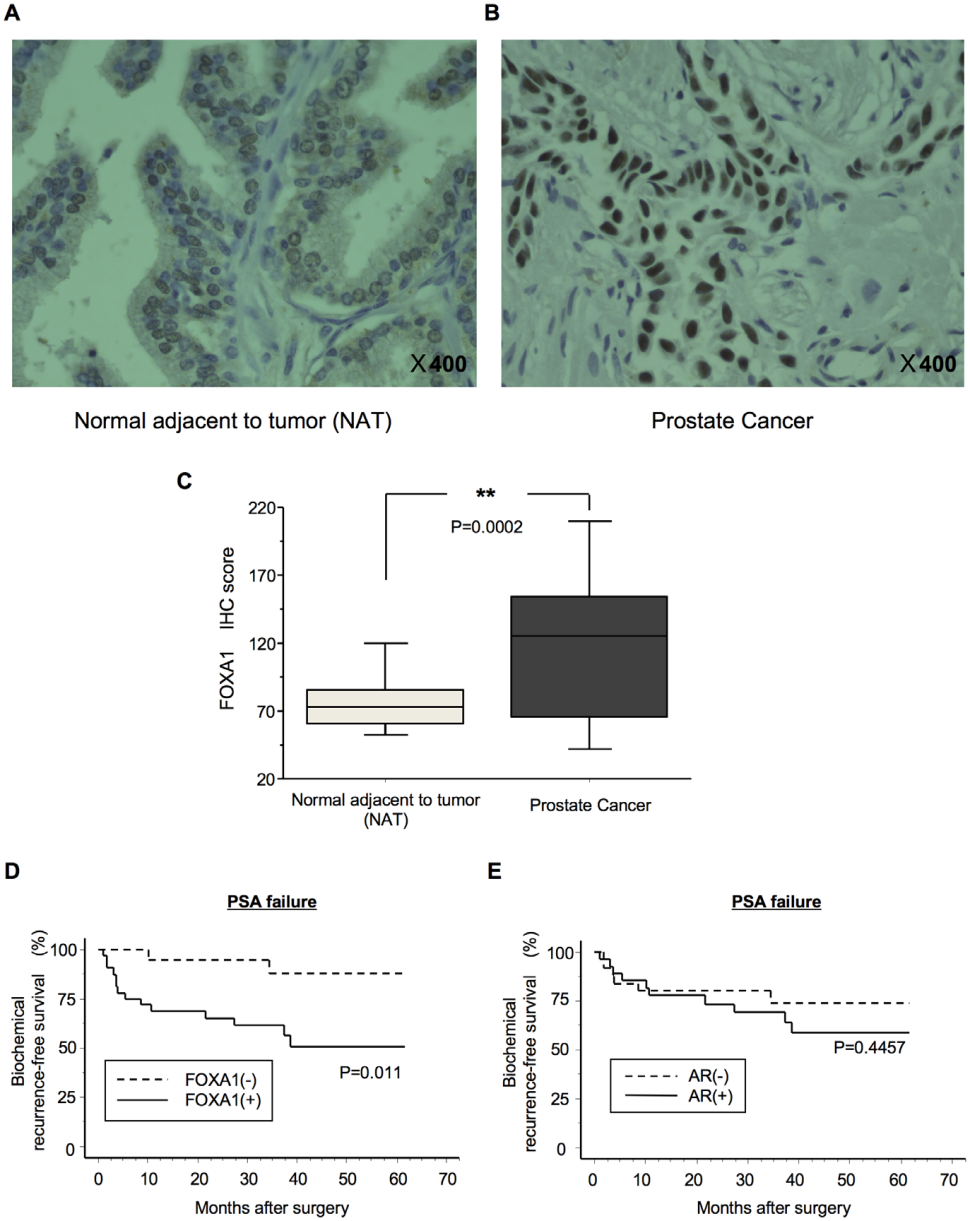
Clinical significance of FOXA1 in prostate cancer (PC). The protein expression of FOXA1 in representative normal adjacent to tumor (NAT) tissue (A) and PC tissue (B), as analyzed using IHC, is shown. NAT displayed low FOXA1 protein expression. FOXA1-positive staining was observed in cases of PC. Positive FOXA1 immunostaining was detected in the nucleus. Original magnification, ×400. (C) Quantification of FOXA1 staining of NAT and PC tissues. The FOXA1 IHC scores were calculated as follows: IHC score  = 1×(number of weakly stained cells in the field)+2×(number of moderately stained cells in the field)+3×(number of intensely stained cells in the field). The FOXA1 IHC scores for normal prostate tissues and for PCs ranged from 30.2 to 123.0 (median, 73.3) and from 20.4 to 260.1 (median, 125.1), respectively. Up-regulated FOXA1 expression (D) was significantly associated with PSA failure (P = 0.011) but up-regulated AR expression (E) was not (P = 0.4457). Log-rank statistics were used to test the difference in survival times between the groups. FOXA1 (+), up-regulated FOXA1; FOXA1 (–), down-regulated FOXA1. AR (+), up-regulated AR; AR (–), down-regulated AR. **indicates a statistical difference of P<0.01.

We investigated the protein expression of FOXA1 in PC by immunohistochemical analysis (IHC) of PC specimens. Representative IHC results of FOXA1 protein expression in normal adjacent to tumor (NAT) and in PC tissues are shown in Figure 1A and B. Positive FOXA1 immunoreactivity was detected in the nucleus and in part of the cytoplasm. A strong FOXA1 immunoreaction was detected in the nuclei of cells in the cancer lesion, whereas cells in the NAT showed weak immunostaining mainly at the cytoplasm. The IHC scores of FOXA1 staining of NAT and of PC lesions ranged from 30.2 to 123.0 (median, 73.3) and from 20.4 to 260.1 (median, 125.1), respectively. The IHC scores of FOXA1 staining of PC lesions were significantly higher than those of NAT (Figure 1C, P = 0.0002). By IHC score, 62% of PC samples were positive for FOXA1, while remaining 38% of samples were negative. The correlations between the clinicopathological characteristics of patients with PC and the status of FOXA1 protein expression using the IHC scoring system are shown in Table 1. These data showed that FOXA1-positive PCs were correlated with the PSA value (P = 0.016), the Gleason score of PCs (P = 0.031) and the expression of AR (P = 0.002) (Table 1). PCs that were positive for both FOXA1 and AR had significantly higher Gleason scores than PCs that were both FOXA1- and AR-negative (P = 0.027) (Table 2).

**Table 1:** Correlation between FOXA1 and clinical factors.

Clinical classification		Total	FOXA1 (−)		FOXA1 (+)		*P-value*
**Age at surgery**							
	<60	11	5	45%	6	55%	0.776
	60–70	30	11	37%	19	63%	
	>70	11	4	36%	7	64%	
**PSA**	median	9.63	7.59		13		0.016*
	(range)	(1.2–31.7)	(1.2–27.8)		(3.8–31.7)		
**T-stage**							
	pT2	32	15	47%	17	53%	0.063
	≥pT3	20	5	25%	15	75%	
**Gleason score**							
	≤6	9	5	56%	4	44%	0.031*
	7	21	10	48%	11	52%	
	≥8	22	5	23%	17	77%	
**AR expression**							
	AR (−)	25	15	60%	10	40%	0.002**
	AR (+)	27	5	19%	22	81%	
	Total	52	20	38%	32	62%	

* P<0.05; **P<0.01. FOXA1(+), FOXA1-positive case; FOXA1(−), FOXA1-negative case.

**Table 2:** FOXA1 and AR expression in prostate cancer specimens.

Specimen	(n)	Gleason score	P-value
**FOXA1 (−) AR (−)**	15	7.07	
**FOXA1 (−) AR (+)**	5	7.4	0.578
**FOXA1 (+) AR (−)**	10	7.7	0.171
**FOXA1 (+) AR (+)**	22	7.86	0.027*

* P<0.05.

Survival analysis using the Kaplan-Meier method showed that FOXA1 up-regulation was a significant factor in PSA failure (Figure 1D; log-rank test, P = 0.011) compared with AR up-regulation (Figure 1E; log-rank test, P = 0.4457). The PSA failure survival rates of cases with up-regulated and down-regulated FOXA1 were 50.7 and 87.7%, respectively. The PSA failure survival rates in cases with up-regulated and down-regulated AR were 58.5 and 73.8%, respectively.

### Evaluation of FOXA1 Protein Expression in PC-derived Cell Lines

**Figure 2:**
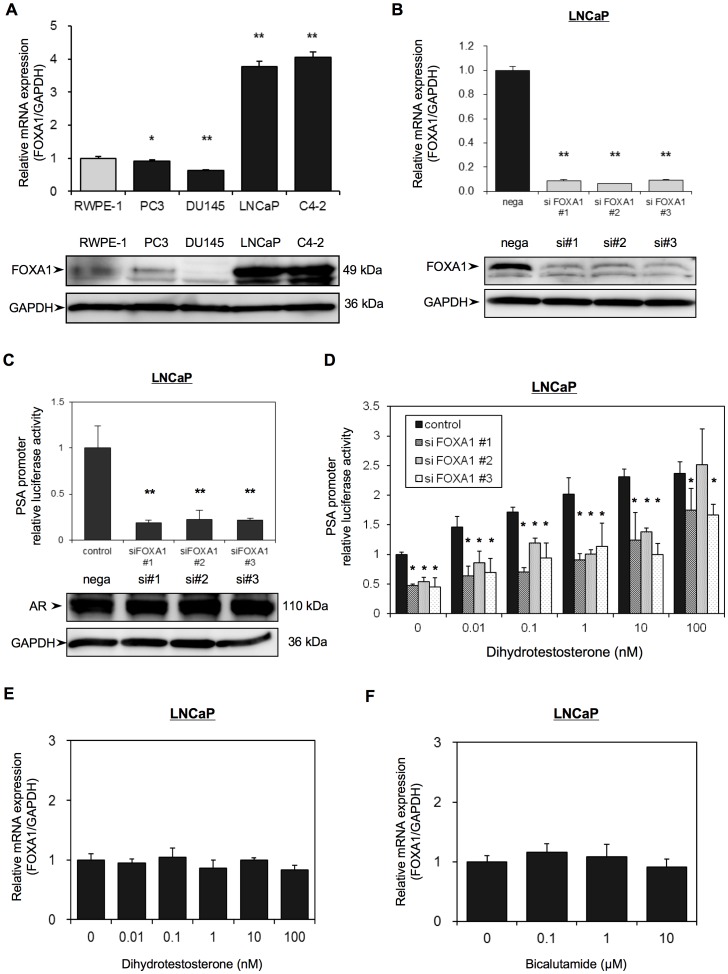
FOXA1 regulates AR activity in LNCaP cells. (A) The mRNA expression and the protein expression of FOXA1 in four PC-derived cell lines (PC-3, DU-145, LNCaP, C4-2) and in non-tumorigenic prostate cell line (RWPE-1) were analyzed using qRT-PCR and Western blotting, respectively. (B) LNCaP cells were transfected with a negative control siRNA (nega) or with one of three different FOXA1 siRNAs (siFOXA1#1–3). After 48 h transfection, RNA was extracted and FOXA1 mRNA was analyzed using qRT-PCR analysis. FOXA1 mRNA expression is expressed relative to GAPDH mRNA expression in the same cell. The results shown are the means ± S.E.M. of three independent experiments. Proteins were extracted 72 h after transfection and FOXA1 and GAPDH protein expression was examined by Western blot analyses. (C, D) LNCaP cells were transfected with the pGL3-PSAp 5.8 luciferase reporter plasmid and with one of three different FOXA1 siRNAs. (#1–3) and were then grown in Phenol red–free RPMI-1640 medium containing 5% CS-FBS. (C) After 72 h transfection, luciferase activities were measured using the Dual-luciferase reporter assay system. Luciferase activities are expressed relative to the control, which was assigned a value of 1. Proteins were extracted 72 h after transfection and the expression of AR and GAPDH was examined by western blot analysis. (D) The transfected cells were incubated with the indicated concentrations of dihydrotestosterone for 72 h, and luciferase activities were measured as in (C). (E, F) Non-transfected LNCaP cells were treated with the indicated concentrations of dihydrotestosterone (E) or bicalutamide (F) for 48 h and FOXA1 mRNA expression was then analyzed using qRT-PCR. FOXA1 mRNA expression is expressed relative to GAPDH mRNA expression in the same cell. Values shown are the means ± SD from at least 3 independent experiments, each performed in triplicate. * and ** indicate statistical difference of P<0.05 and P<0.01, respectively.

We next investigated FOXA1 mRNA and protein expression in PC by quantitative reverse transcription-polymerase chain reaction (qRT-PCR) analysis and Western blotting respectively, of four PC-derived cell lines, including androgen independent cell line (PC-3 and DU145), androgen sensitive cell line (LNCaP), androgen insensitive cell line (C4-2) established from castrated host of LNCaP and non-tumorigenic prostate cell line (RWPE-1) [16]–[19] (Figure 2A). The molecular weight of FOXA1 by Western blotting was 49 kDa. Both FOXA1 mRNA and protein expression were significantly higher in the androgen-sensitive LNCaP cells and C4-2 cells than in the other cell lines.

To obtain cells in which FOXA1 expression was transiently knocked down, we transfected LNCaP cells with one of three different FOXA1 siRNAs (siFOXA1#1–3) plasmids or with a negative control siRNA (nega) plasmid. We then analyzed FOXA1 mRNA and protein expression in these siFOXA1-transfected cells using qRT-PCR and Western blot analyses, respectively. SiFOXA1-transfected cells shows reduction of FOXA1 mRNA level by almost 90% compared to that in nega-transfected cells (nega). FOXA1 protein levels in siFOXA1-transfected cells were also strongly decreased compared with nega-transfected cells (Figure 2B).

### The Effect of FOXA1 on Androgen Receptor Activity and the Effect of AR Activation on FOXA1 Expression

To determine the effect of FOXA1 knockdown on AR activity, we transfected the siFOXA1-transfected LNCaP cells with a luciferase expression plasmid driven by the prostate-specific antigen (PSA) promoter, pGL3PSAp-5.8. Although there was no difference in the protein expression of AR in siFOXA1-transfected cells or in nega-transfected cells, siFOXA1-transfected cells showed almost 80% reduction of PSA promoter activity compare to that of nega-transfected cells (Figure 2C). When control LNCaP cells that were only transfected with pGL3PSAp-5.8 were treated with the AR ligand dihydrotestosterone (DHT), PSA promoter activity was increased in a dose-dependent manner. However, co-transfection of these cells with siFOXA1 significantly reduced PSA promoter activity following treatment with various concentrations of DHT (Figure 2D). To look at the influence of FOXA1depletion on endogenous PSA expression, as FOXA1 acts to remodel chromatin, we analyzed PSA mRNA expression in siFOXA1-transfected cells using qRT-PCR. Figure S1A shows that FOXA1 depletion mediated significant reduction of PSA mRNA level compared to that in nega-transfected cells.

To determine whether FOXA1 mRNA expression is mediated by AR activity, we tested the effect of treatment of LNCaP cells with DHT and or with the AR antagonist bicalutamide on FOXA1 mRNA expression. QRT-PCR analysis showed that there was no difference in the mRNA expression of FOXA1 in androgen-sensitive LNCaP cells following growth in medium containing DHT or in medium containing bicalutamide (Figure 2E and F). These results indicated that FOXA1 mediates activation of AR, however, expression of FOXA1 itself is not regulated by androgen.

### FOXA1 Regulates Cell Proliferation in PC Cells

**Figure 3:**
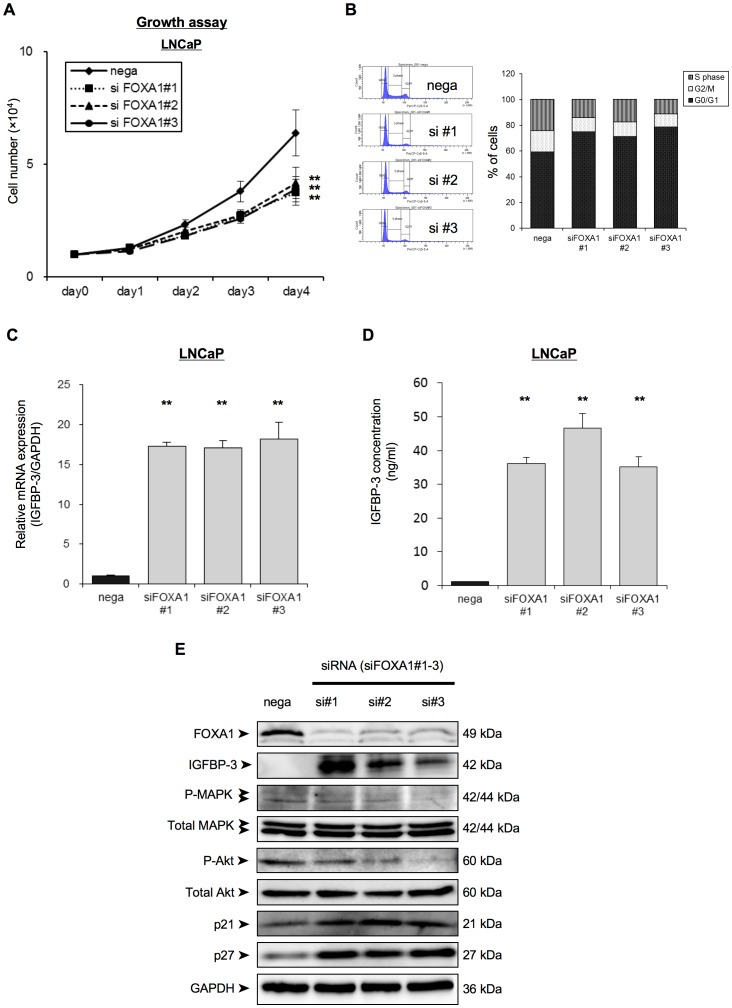
FOXA1 regulates PC cell proliferation in IGFBP-3 dependent manner. (A) LNCaP cells were transfected with a negative control siRNA (nega) or with one of three different FOXA1 siRNAs (#1–#3), and were then reseeded into a 24-well culture plate. Cell proliferation was then assayed over 4 days by counting the number of cells for each transfection condition, as indicated. (B) The cell-cycle stage of the cells was analyzed using propidium iodide staining and FACS analysis 24 h after siRNA transfection and the percentage of cells in each transfected population that was in each cycle phase was calculated. (C) LNCaP cells were transfected with a negative control siRNA (nega) or with one of three different FOXA1 siRNAs (siFOXA1#1–3). After 48 h of transfection IGFBP-3 mRNA expression was analyzed by qRT-PCR. IGFBP-3 mRNA expression is expressed relative to GAPDH mRNA expression in the same cell. (D) LNCaP cells were transfected with a negative control siRNA (nega) or with one of three different FOXA1 siRNAs (siFOXA1#1–3). After 72 h of transfection, IGFBP-3 concentration of the media was analyzed by ELISAs. (E) Proteins were extracted 72 h after transfection and the expression of IGFBP3, of the insulin-receptor signaling related proteins (total amount of MAPK, phospho-MAPK, total amount of Akt and phospho-Akt), of G0/G1 arrest-related cell cycle regulatory proteins (p21cip1, p27kip1) and of GAPDH was examined by western blot analysis. Protein molecular weights are indicated at right sides of the panel. These results are the means ± S.E.M. of three independent experiments. **indicates a statistical difference of P<0.01.

To investigate the effect of FOXA1 knockdown on cell proliferation, the cell growth of siFOXA1-transfected LNCaP cells was monitored over 4 days. The siFOXA1-transfected cells showed a significant decrease in cellular growth compared with nega-transfected cells (Figure 3A). On the other hand, FOXA1 overexpressing cells significantly increased cellular growth compared with nega-transfected cells (Figure S1C). To investigate effect of FOXA1 in cell cycle progression, we analyzed the cell cycle stages of siFOXA1-transfected cells using flow cytometric analysis. The percentage of siFOXA1-transfected cells in the G0/G1 phase was higher than that of nega-transfected cells (Figure 3B), suggesting that down-regulation of FOXA1 inhibited cellular proliferation by induction of G0/G1 arrest.

### Depletion of FOXA1 Regulates IGFBP-3 and IGF Signaling

To gain an insight into genes that are up-regulated by FOXA1 depletion of PC cells, we performed gene expression microarray analysis of cells depleted of FOXA1 (Table 3 and 4). We identified 421 genes that were more strongly expressed in FOXA1-depleted cells than in the negative control transfectants. Clustering of the up-regulated genes into functional categories indicated that the highest proportion of up-regulated genes were related to cell proliferation, followed by response to wounding, muscle system processes, defense responses, inflammatory responses, negative regulation of cell proliferation, response to hypoxia and cell maturation (Table 3). The up-regulated genes that are related to negative regulation of cell proliferation are listed in Table 4. Of these up-regulated genes, we further studied insulin-like growth factor binding protein 3 (IGFBP-3), since this gene is a well-established tumor-suppressive gene in PC [20]–[23].

**Table 3:** Functional Annotation Chart of 421 genes that were upregulated in FOXA1-depleted cells.

Term	Gene count	%	P-value	Benjamini
Regulation of cell proliferation	43	11.2	1.87E-08	4.05E-05
Response to wounding	31	8.07	7.10E-07	7.69E-04
Muscle system process	16	4.17	2.43E-06	0.001755
Acute phase	7	1.82	1.30E-05	0.005513
Defense response	31	8.07	1.42E-05	0.007665
Inflammatory response	21	5.47	1.67E-05	0.007216
Negative regulation of cell proliferation	22	5.73	2.39E-05	0.008614
Response to hypoxia	13	3.39	2.42E-05	0.007479
Cell maturation	10	2.6	2.53E-05	0.006849
Regulation of system process	20	5.21	2.76E-05	0.00664
Response to oxygen levels	13	3.39	4.02E-05	0.008689
Positive regulation of cellular biosynthetic process	32	8.33	4.38E-05	0.008594
Di-, tri-valent inorganic cation homeostasis	17	4.43	4.38E-05	0.007883
Developmental maturation	11	2.86	4.92E-05	0.008173
Extracellular space	31	8.07	5.68E-05	0.017412
Positive regulation of biosynthetic process	32	8.33	5.71E-05	0.008802

**Table 4:** Genes involved in negative regulation of cell proliferation that were up-regulated in FOXA1-depleted cells.

Entrez gene ID	Gene symbol	Gene name	Log2 ratio
NM_000165	GJA1	gap junction protein, alpha 1, 43 kDa	5.5
NM_001013398	IGFBP3	insulin-like growth factor binding protein 3	3.91
NM_002423	MMP7	matrix metallopeptidase 7 (matrilysin, uterine)	3.23
NM_005596	NFIB	nuclear factor I/B	2.37
NM_000660	TGFB1	transforming growth factor, beta 1	2.29
NM_000599	IGFBP5	insulin-like growth factor binding protein 5	2.28
NM_000576	IL1B	interleukin 1, beta	1.98
NM_203339	CLU	clusterin	1.98
NM_001955	EDN1	endothelin 1	1.94
NM_001956	EDN2	endothelin 2	1.82
NM_032551	KISS1R	KISS1 receptor	1.59
NM_005655	KLF10	Kruppel-like factor 10	1.54
NM_000076	CDKN1C	cyclin-dependent kinase inhibitor 1C (p57, Kip2)	1.51
NM_000316	PTH1R	parathyroid hormone 1 receptor	1.38
NM_001904	CTNNB1	catenin (cadherin-associated protein), beta 1, 88 kDa	1.06
NM_002133	HMOX1	heme oxygenase (decycling) 1	1.03

To confirm the up-regulation of IGFBP-3 in LNCaP cells by FOXA1 depletion that was indicated by the microarray data, we analyzed IGFBP-3 mRNA and protein expression in LNCaP cells transfected with siFOXA1 plasmids or with the negative control siRNA plasmid, using qRT-PCR and Western blot analyses, respectively. Figure 3C shows that IGFBP-3 mRNA expression in siFOXA1-transfected cells was significantly higher than that in nega-transfected cells. IGFBP-3 protein levels in siFOXA1-transfected cells were also strongly increased compared with the level in nega-transfected cells (Figure 3E). Moreover, to evaluate changes in the levels of secreted IGFBP-3, we performed ELISAs. Figure 3D shows that IGFBP-3 concentration in the media of siFOXA1-transfected cells was also strongly increased (over 30 times) compared with that of nega-transfected cells. Since IGFBP-3 modulates IGF-1 signaling, we next investigated if FOXA1 regulation of IGFBP-3 expression was associated with modulation of the expression level or activation status of IGF-1 downstream signals in these cells (Figure 3E). Western blot analysis indicated marked up-regulation of IGFBP-3, down-regulation of the phosphorylation of the MAPK and Akt, and up-regulation of the protein expression of the cyclin-dependent kinase inhibitors (p21cip1, p27kip1) in siFOXA1-transfected cells compared to nega-transfected cells (Figure 3E). Furthermore, FOXA1 depletion significantly blocked the activation of AKT in response to IGF-1 treatment in LNCaP (Figure S1E). These results showed that FOXA1 regulation of IGFBP-3 is accompanied by regulation of IGF-1 downstream signals.

**Figure 4:**
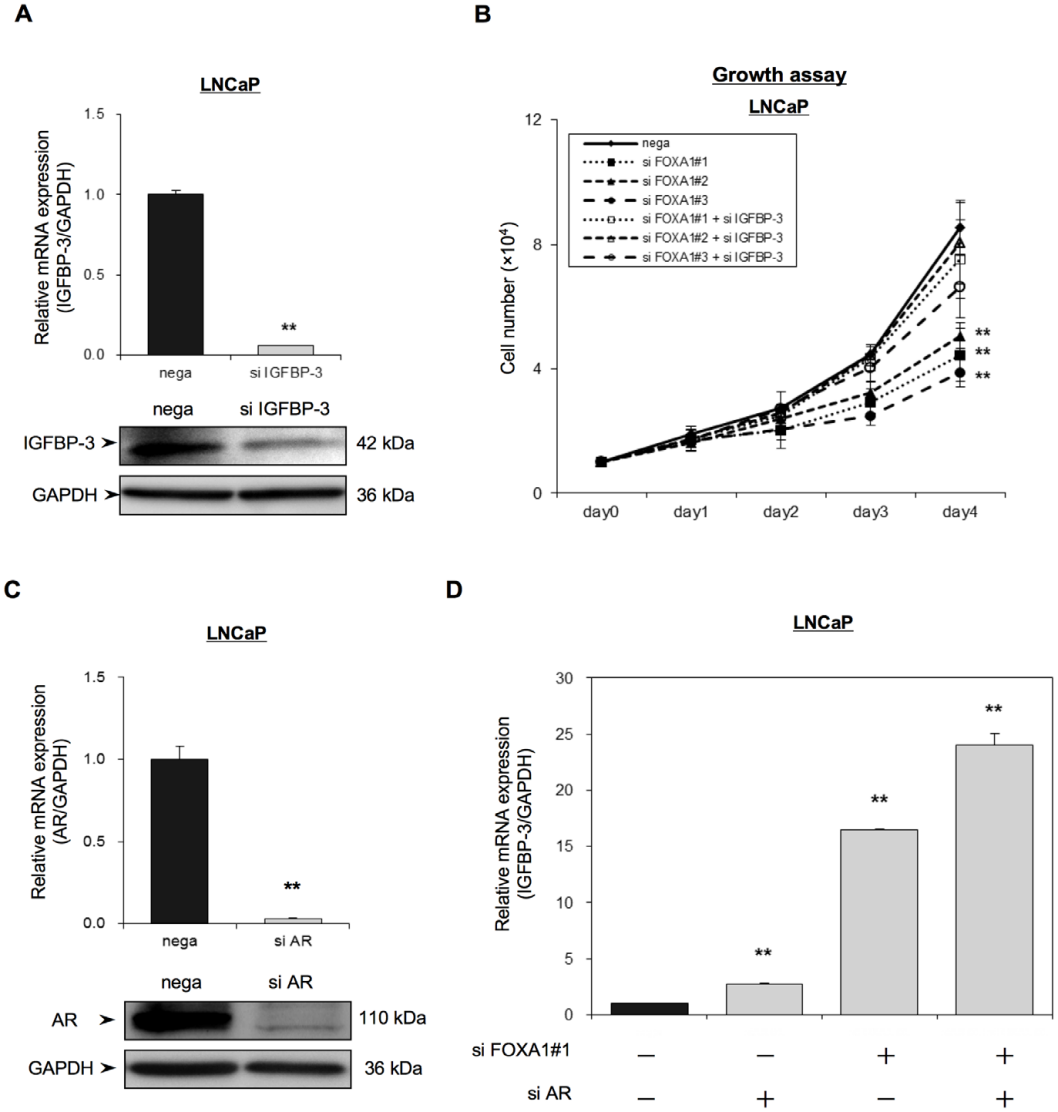
Regulation of IGFBP-3 expression and AR activity through FOXA1. (A) LNCaP cells were transfected with a negative control siRNA (nega) or with an IGFBP-3 siRNA (siIGFBP-3) and, 48 h later, IGFBP-3 mRNA and protein levels were analyzed using qRT-PCR and Western blot analysis. IGFBP-3 mRNA expression is expressed relative to GAPDH mRNA expression in the same cell. (B) LNCaP cells were transfected with a negative control siRNA (nega) or with one of three FOXA1 siRNAs (siFOXA1#1–#3) and/or an IGFBP-3 siRNA (siIGFBP-3), and were reseeded into a 24-well culture plate. Cell proliferation was measured over 4 days and the number of cells for each transfected population is indicated. (C, D) LNCaP cells were transfected with a negative control siRNA (nega) or with an AR siRNA (si AR) and/or a FOXA1 siRNA (si FOXA1#1) and, 48 h after transfection, AR mRNA and protein (C) or IGFBP-3 mRNA levels (D) were analyzed using qRT-PCR and/or western blot analysis. The mRNA levels are expressed relative to GAPDH mRNA expression in the same cell. The results shown are the means ± S.E.M. of three independent experiments. ** indicate statistical difference of P<0.01.

In order to determine the importance of IGFBP-3 for FOXA1 regulation of PC proliferation, we compared the growth of siFOXA1-transfected LNCaP cells and that of siIGFBP-3-transfected cells. IGFBP-3 was transiently knocked down by transfection of LNCaP cells with the IGFBP-3 siRNA (siIGFBP-3) plasmid and IGFBP-3 mRNA and protein expression in the siIGFBP-3-transfected cells was assessed using qRT-PCR and western blot analysis, respectively. Figure 4A shows that IGFBP-3 mRNA and protein expression in the siIGFBP-3-transfected cells was significantly lower than that in the nega-transfected cells. Although the siFOXA1-transfected cells showed a significant decrease in cellular growth compared with nega-transfected cells (Figure 3A and 4B), the anti-proliferative effect of siFOXA1 was reversed by dual transfection of siFOXA1 and siIGFBP-3 (Figure 4B). This result indicated that FOXA1 regulates cell proliferation in an IGFBP-3-dependent manner.

Since activation of AR has been reported to down-regulate IGFBP-3 expression [24], we further studied the effect of AR on FOXA1 mediated regulation of IGFBP-3 using siAR-transfected cells (Figure 4C and 4D). Depletion of AR did increase IGFBP-3 mRNA expression (2.8 times). However, the increase in IGFBP-3 expression observed in siFOXA1-transfected cells was much higher (16.4 times) than that in siAR-transfected cells. Moreover, simultaneous depletion of AR and FOXA1 resulted in a further increase in IGFBP-3 expression (24.0 times) compared with depletion of FOXA1 alone, suggesting that FOXA1 and AR cooperatively regulate IGFBP-3 expression (Figure 4D).

### Role of FOXA1 on Castration Resistant Prostate Cancer (CRPC) Models

**Figure 5:**
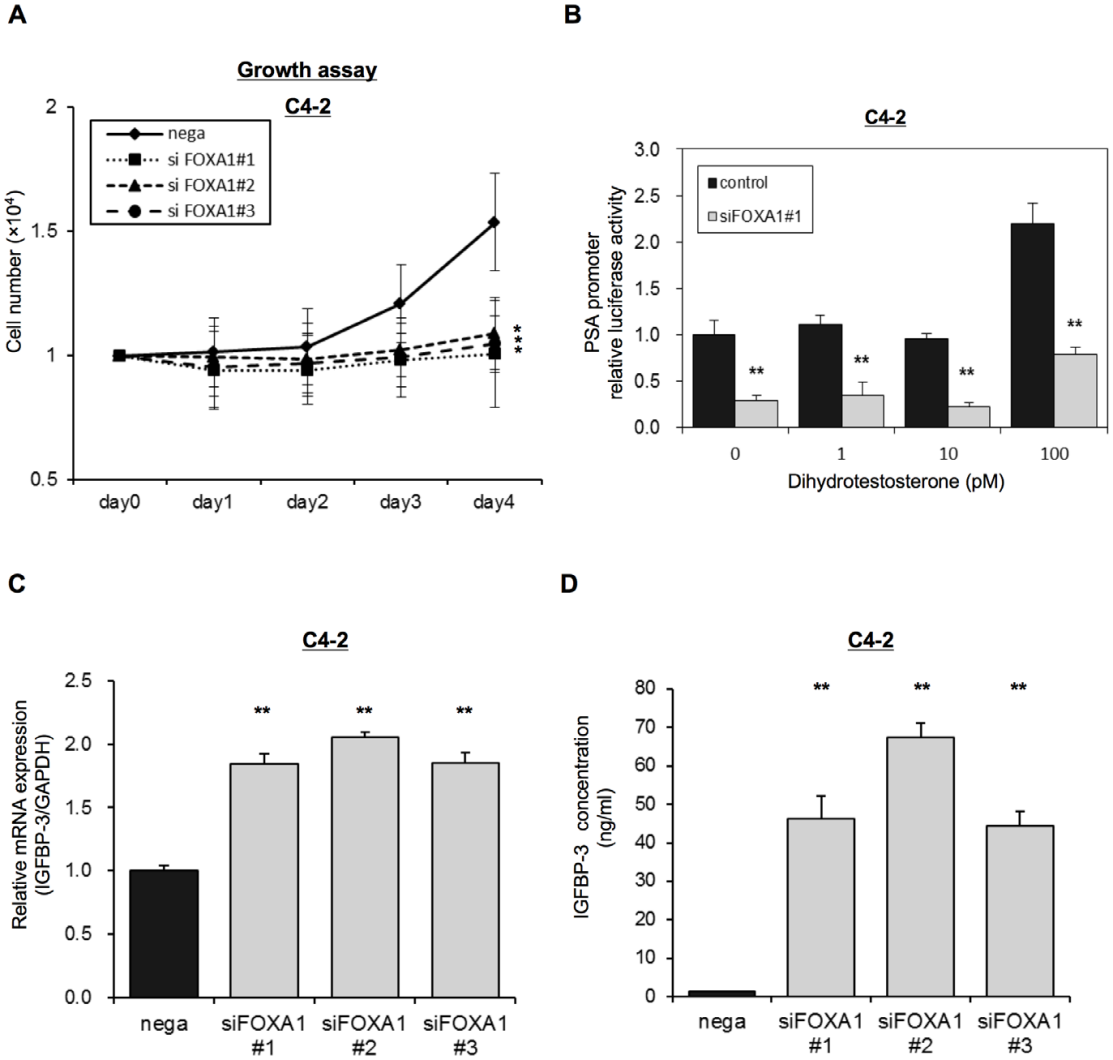
FOXA1 also regulates C4-2 cell proliferation in IGFBP-3 dependent manner. (A) C4-2 cells were transfected with a negative control siRNA (nega) or with one of three FOXA1 siRNAs (#1–#3) and were then reseeded into a 24-well culture plate with Phenol red–free RPMI-1640 medium containing 5% CS-FBS. Cell proliferation was then measured over 4 days and is shown as the number of cells for each transfected population. (B) C4-2 cells were transfected with the pGL3-PSAp 5.8 luciferase reporter and with a FOXA1 siRNA (#1) and were grown in Phenol red–free RPMI-1640 medium containing 5% CS-FBS with the indicated concentrations of dihydrotestosterone (1–100 pM). Following 72 h transfection, luciferase activity was measured using the Dual-luciferase reporter assay system. (C) C4-2 cells were transfected with a negative control siRNA (nega) or with one of three different FOXA1 siRNAs (siFOXA1#1–3). After 48 h of transfection IGFBP-3 mRNA expression was analyzed by qRT-PCR. IGFBP-3 mRNA expression is expressed relative to GAPDH mRNA expression in the same cell. (D) C4-2 cells were transfected with a negative control siRNA (nega) or with one of three different FOXA1 siRNAs (siFOXA1#1–3). After 72 h of transfection, IGFBP-3 concentration of the media was analyzed by ELISAs. The results shown are the means ± S.E.M. of three independent experiments. * and ** indicate statistical difference of P<0.05 and P<0.01, respectively.

We assessed the role of FOXA1 on androgen-independent prostate cancer using C4-2 cells, established from castrated host of LNCaP cells. Depletion of FOXA1 significantly inhibited cell growth of C4-2 cells even at the low-androgen environment (5% charcoal filtered FCS) compare with nega-transfected cells (Figure 5A). Overexpresssion of FOXA1 in C4-2 cells significantly promoted cell growth (Figure S1D). When control C4-2 cells were treated with low-doses of DHT (1–100 pM), PSA promoter activity was up-regulated at a DHT concentration of 100 pM. However, transfection of FOXA1 siRNA blocked the up-regulation of PSA promoter activity that occurred in response to the DHT treatment at 100 pM in C4-2 cells (Figure 5B). Similarly, depletion of FOXA1 significantly inhibited PSA mRNA expression in qRT-PCR (Figure S1B). We also analyzed IGFBP-3 mRNA expression in C4-2 cells transfected with siFOXA1 plasmids or with the negative control, using qRT-PCR. Figure 5C shows that IGFBP-3 mRNA expression in siFOXA1-transfected cells was significantly higher than that in nega-transfected cells. Figure 5D shows that IGFBP-3 concentration in the media of siFOXA1-transfected cells were also strongly increased (over 40 times) compared with the level in nega-transfected cells. siFOXA1 significantly inhibited phosphorylation of Akt after stimulating with IGF-1 in C4-2 cells similar to that of LNCaP cells, indicating negative regulation of IGF-1 signaling upon FOXA1 depletion (Figure S1F).

## Discussion

The present study investigated the role of FOXA1 in PC progression. We identified the tumor suppressor IGFBP-3 as a novel target of FOXA1, and showed that FOXA1 targeting of IGFBP-3 plays an essential role in regulating PC cell proliferation.

IGFBP-3 has been known to function as a tumor suppressor or a cell cycle inhibitor in PC cells [21], [24]. Increased IGFBP-3 expression upon FOXA1 depletion inhibited the phosphorylation of the MAPK and of Akt and mediated cell cycle arrest through increased expression of the cell cycle regulators p21 and p27 in PC cells. FOXA1 has recently been reported to regulate cell cycle and cell proliferation in LNCaP cells [15]. Our data indicated that IGFBP-3 can be a main regulator of FOXA1-dependent cell proliferation in LNCaP cells. Based on our results, not only the AR but also IGFBP-3 may be a key factor in FOXA1 mediated signaling in PC.

FOXA1 is a transcription factor that modulates the function of steroid hormone receptors. In the prostate, FOXA1 interacts with the AR and controls androgen induced activation of prostate-specific genes [6]. Recently, AR and adjacent FOXA1 binding sites have been are found on multiple promoters in prostatic cells [7], [25]. Similar to the function of AR, FOXA1 expression is necessary for normal development as well as for androgen regulation of PC [26]. Increased expression of FOXA1 has been observed at all stages of prostate development in a mouse model [27]. We found that FOXA1 was overexpressed in the nuclei of PC cells, and that this overexpression was associated with increased PSA, Gleason Scores and AR expression. Furthermore, the overexpression of FOXA1 was a significant factor in PSA failure. Those result are consistent with previous report indicating that FOXA1 staining intensity was significantly weaker in the adjacent non-cancerous than cancerous tissue [28] and that strong FOXA1 staining associated with poor prognosis, higher pT stages and increased Gleason Grade [15], [28]. A high level of FOXA1 was previously found in 89% of metastatic PC and the expression of FOXA1 was positively correlated with tumor size, extraprostatic invasion, AR and lymph node invasion [29]. FOXA1 contributes to the androgen-dependent growth of PC [30]. FOXA1 also plays an essential role in expression of ubiquitin-conjugating enzyme E2C (UBE2C), a gene that inactivates the M-phase checkpoint, and UBE2C is over-expressed in androgen-independent PC cell lines and clinical cases [31]. Genome-wide association studies indicated the presence of a single nucleotide polymorphism (rs119986220) in the FOXA1 binding site within 8q24 alleles that is associated with PC risk [25]. The combined evidence supports the connection between FOXA1 and tumor progression in PC.

Through gene expression analysis, we identified IGFBP-3 as a down-stream target of FOXA1. IGFBP-3 is an insulin-like growth factor binding protein whose main function is associated with IGF delivery and availability [32]. IGFBP-3 has a greater affinity for IGF-1 than the IGF-1 receptor and blocks the IGF-1 mediated pathway. IGFBP-3 can therefore reduce IGF-1 bioavailability and inhibit IGF-1 interaction with the IGF-1 receptor, thereby functioning as a tumor suppressor [21], [24]. An IGF-independent role of IGFBP-3 has also recently been identified. IGFBP-3 binds to the transforming growth factor-β type V receptor and thereby inhibits cell growth independently of its effects on IGF [20], [33]. Induction of apoptosis through synergistic interaction of IGFBP-3 with a nuclear receptor such as retinoid X receptor (RXR)-alpha has also been observed in PC cells [24], [34]. Our result also indicated that increased expression of IGFBP-3 upon depletion of FOXA1 significantly inhibited PC proliferation in androgen-sensitive LNCaP cells, most likely through the IGF-1 signaling pathway. Increased IGFBP-3 upon FOXA1 depletion inhibited phosphorylation of signaling mediators in the IGF-1 signaling pathway including MAPK and Akt, and mediated cell cycle arrest through p21 and p27 in PC cells. The latter results are consistent with a previous report that FOXA1 was up-regulated in a thyroid cancer lesion and that depletion of FOXA1 induced cell cycle arrest and a reduction in cell proliferation via a p27-dependent mechanism [14].

Acquisition of androgen resistance in PC, which is termed castration resistance, is a major problem that limits the patient’s quality of life as well as life expectancy. Although a number of mechanisms have been proposed to explain the development of castration-resistant PC (CRPC), the major pathway of this resistance still appears to involve the AR, even during anti-androgen therapy. Amplification of the AR, mutation of the AR, overexpression of AR coactivators or deregulation of growth factors/cytokines may all occur in CRPC and induce a change in the response to AR antagonists. Another mechanism of CRPC is through escape from apoptosis due to the loss of the tumor-suppressive gene PTEN and suppression of the anti-apoptotic gene BCL-2 [35]. Another known cause of CRPC is that the AR can be activated in human PC cells in the absence of androgens by IL-6 [36], [37]. Existence of so many complex pathways associated with the regulation of AR activation makes it hard to control growth of CRPC. Based on our data, the targeting of FOXA1 may provide a practical approach for the down-regulation of AR activity in CRPC. Depletion of FOXA1 significantly down-regulated ligand-dependent AR activation (up to 80%) in LNCaP cells and C4-2 cells. Moreover, depletion of FOXA1 up-regulated IGFBP-3 expression and inhibited IGF-1 signaling, which is a major pathway for ligand-independent activation of AR [38]. However, another line of evidence indicated dual role of FOXA1. Although FOXA1 serves as a pioneer factor promoting AR and chromatin interaction, it also creates corepressor complexes inhibiting AR and chromatin binding. Thus FOXA1 depletion results in appearance of a large number of new ARBs which linked to androgen regulation of nearby genes [28], [39]. Since FOXA1 mediated regulation of AR is not fully characterized, further analysis will reveal the therapeutic significance of FOXA1 in CRPC.

As the next step, in determination of FOXA1 function, we are currently studying the precise mechanisms by which FOXA1 regulates IGFBP-3 expression. Since FOXA1 is a transcription factor, we are currently determining whether a FOXA1 binding site exists within the promoter region of IGFBP-3 that may control IGFBP-3 expression. Another possible mechanism of FOXA1 regulation of IGFBP-3 could be through FOXA1 regulation of the AR. The AR binds to androgen responsive elements in the distal region of the IGFBP-3 promoter [32]. Activation of the AR with DHT has been reported to down regulate IGFBP-3 expression, and this down-regulation was reversed by application of the anti-androgen bicalutamide [21], [24]. Although depletion of FOXA1 did not affect the expression level of AR, the binding of FOXA1 to AR may affect the affinity of AR binding to the promoter region of IGFBP-3. Our data indicated that the increased expression of IGFBP-3 mediated by depletion of FOXA1 was significantly higher than that mediated by depletion of AR. Depletion of AR together with FOXA1 in LNCaP cells induced a moderate increase in IGFBP-3 expression compared with single depletion of FOXA1 (Figure 4D). These data may indicate that the expression of IGFBP-3 is cooperatively regulated by AR and FOXA1. Further study will clarify the precise mechanism by which FOXA1 regulates IGFBP-3.

Another direction of future study will be evaluation of the therapeutic effect of FOXA1 against PC, especially against CRPC. Current therapy mainly targets the AR by lowering androgen concentrations or by blocking the ligand binding domain of the AR. Nevertheless, resistance to therapy still occurs. One of the potentially therapeutically useful features of FOXA1 is that it does not require the ligand binding domain of the AR for its effects on the AR. FOXA1 directly binds to the DNA binding domain of AR and alters the 3-D structure of the AR [5]. Therefore, suppression of FOXA1 may be an effective therapy for blocking AR activity in cases where a C-terminus deleted splicing variant of AR or an AR that is mutated within the ligand binding domain is expressed. Such AR variants have been observed in CRPC patients [40].

In summary, our findings support a regulatory role for FOXA1 in tumor proliferation via the regulation of IGFBP-3 expression in PC. Further dissection of the contribution of FOXA1 to dictation of tumor cell and metastatic outcome, and of its mechanism of action, may provide new opportunities for therapeutic targeting of advanced stages of PC.

## Materials and Methods

### Ethics Statement

All patients provided informed consent for a protocol that was reviewed and approved by the institutional review board of Chiba University. Written informed consent was obtained from all patients.

### Reagents and Antibodies

Lipofectamine 2000 and Lipofectamine™ RNAiMax reagent, siRNAs siFOXA1 (#1–3) (Stealth Select RNAi™ siRNA; HSS104878, HSS104880 and HSS179280, respectively), siIGFBP-3 (Stealth Select RNAi™ siRNA, HSS105267), siAR (Stealth Select RNAi™ siRNA, HSS100619) and negative universal control siRNA (Stealth™ RNAi) were purchased from Invitrogen (Carlsbad, CA, USA). Anti-FOXA1 (C-20) and anti-IGFBP-3 antibodies (H-98) were obtained from Santa Cruz Biotech Inc. (Santa Cruz, CA, USA). Anti-AR [AR441] (ab9474) was obtained from Abcam. Anti-p21cip1 and anti- p27kip1 were from Becton Dickinson Biosciences (San Diego, CA, USA). Anti-Akt, anti-phosphorylated Akt (Ser473), anti-p44/42 MAPK and anti-phosphorylated p42/44 MAPK were obtained from Cell Signaling Technology (Danvers, MA, USA). Anti-GAPDH was purchased from Applied Biosystems (Foster, CA, USA). The luciferase reporter plasmid, pGL3–5.8PSAp, which is driven by a 5.8-kb PSA promoter that includes androgen-response elements, was kindly provided by Dr. A. Mizokami at Kanazawa University, Ishikawa, Japan. DHT, bicalutamide and IGF-1 were purchased from Sigma-Aldrich Japan K.K. (Tokyo, Japan).

### PC Tissue Specimens

PC tissue samples were obtained from 52 patients who underwent radical prostatectomy for PC at Chiba University Hospital between 2006 and 2010 and from whom pretreatment information was available. Pathological evaluation of these tissues was confirmed. Histopathological analysis of the tissues was performed according to the World Health Organization criteria by the Department of Pathology, Chiba University Hospital. Clinicopathological staging was determined by the TNM classification of the International Union Against Cancer. All patients had PC that was histologically confirmed, and tumor samples were checked to ensure that tumor tissue was present in the specimen.

### Cell Culture and Transfection

PC-3, DU145, LNCaP and C4-2 cell lines, derived from human PCs, as well as the normal human prostate cell line RWPE-1, were obtained from the American Type Culture Collection (Manassas, VA, USA). The PC cell lines were cultured in RPMI-1640 medium supplemented with 10% fetal bovine serum (FBS), and were maintained in an incubator with a humidified atmosphere of 95% air and 5% CO_2_ at 37°C. For steroid-free conditions, Phenol red-free RPMI-1640 medium was used with 5% charcoal-stripped FBS (CS-FBS).

PC cells were transfected with DNA and/or siRNA using Lipofectamine 2000 and/or Lipofectamine™ RNAiMax reagent (Invitrogen), according to the manufacturer’s instructions.

### Evaluation of FOXA1 mRNA Expression

Total RNA was isolated using the RNeasy Mini Kit (Qiagen, Venlo, Netherlands) according to the manufacturer’s instructions. First-strand complementary DNA (cDNA) was synthesized using the ImProm-II™ Reverse Transcription System with random primers (Promega, Tokyo, Japan). QRT-PCR was performed using a LightCycler® 480 Real-Time PCR System (Roche Diagnostics GmbH, Mannheim, Germany). The PCR reactions were carried out in a final volume of 20 µl of a reaction mixture of LightCycler 480 Probe Master (Roche). The initial PCR step consisted of a 5-min hold at 95°C, followed by 45 cycles of 10-sec denaturation at 95°C, 30-sec annealing/extension at 60°C and 1-sec signal acquisition at 72°C. TaqMan® probes and primers for FOXA1 (P/N: Hs00270129_m1), AR (P/N: Hs00171172_m1), IGFBP-3 (P/N: Hs00181211_m1) (Applied Biosystems) and PSA (4410629-T; Roche Diagnostics GmbH, Mannheim, Germany) were used. The transcript amounts for the target genes were estimated from the respective standard curves and were normalized to the amount of the glyceraldehyde-3-phosphate dehydrogenase (GAPDH) (A/N: NM_002046) (Applied Biosystems) transcript that was determined in corresponding samples.

To study the effect of DHT treatment, LNCaP or C4-2 cells were incubated in phenol-red free RPMI-1640 medium containing 5% CS-FBS for 36 h. The cells were then seeded and treated with the indicated concentrations of DHT for 48 h. To study the effect of bicalutamide treatment, LNCaP cells were incubated in RPMI-1640 medium containing 10% FBS with the indicated concentrations of bicalutamide for 48 h. After incubation, total RNA was extracted, cDNA was synthesized and qRT-PCR was performed.

### Luciferase Assay

Luciferase Assay were performed according to the protocol described previously [41]. Briefly, LNCaP or C4-2 cells were seeded at a density of 5×10^4^ cells/well into a 24-well culture plate. After 24 h, the medium was changed to phenol red-free RPMI-1640 medium containing 5% FBS and the cells were treated with the indicated concentrations of DHT without antibiotics. The cells were then transfected with 0.4 mg of the luciferase reporter plasmid, pGL3-PSAp 5.8 and siFOXA1(#1–3) using Lipofectamine 2000 (Invitrogen). Cell lysates were prepared 72 h after transfection and luciferase activities were measured using the Dual-luciferase reporter assay system (Promega).

### Western Blot Analysis

Protein expression was determined by immunoblotting using the specific antibodies as previously described [42]. Protein expression levels were normalized to GAPDH expression. Cells were harvested 72 h after transfection. Cell lysates were prepared using radioimmunoprecipitation assay (RIPA) buffer (150 mmol/L NaCl, 50 mmol/L Tris (pH 8.0), 0.5% deoxycholic acid, 1% NP40 and 1 mmol/L phenyl methyl-sulfonyl fluoride). Protein content was quantified using the bicinchoninic acid protein assay kit (Thermo, Waltham, MA, USA), and protein samples (30 µg) were subjected to SDS-PAGE and transferred to Hybond-C membranes (GE Healthcare, Little Chalfont, UK). Membranes were blocked in 5% milk in TBS-T (TBS containing 0.05% Tween 20; 1 h at room temperature), and, following incubation with the respective primary antibody (1∶100 dilution, overnight at 4°C), membranes were exposed to species-specific horseradish peroxidase-labeled secondary antibodies. Signals were detected using the ECL Plus Western Blotting Detection System (GE Healthcare, Little Chalfont, UK) and were visualized using LAS-4000 mini (Fujifilm, Tokyo, Japan).

### IHC

IHC was performed on 4-µm sections of paraffin-embedded specimens using goat anti-FOXA1 polyclonal antibody (C-20, Santa Cruz Biotechnology). Briefly, after deparaffinization and hydration, the slides were treated with endogenous peroxidase in 0.3% hydrogen peroxide solution in 100% methanol for 30 min, after which the sections were blocked for 2 h at room temperature with 1.5% blocking serum (Santa Cruz) in PBS before reacting with anti-FOXA1 antibody (1∶100 dilution) at 4°C in a moist chamber overnight. Following incubation with the primary antibody, the specimens were washed three times in PBS and were then treated with the Envision reagent followed by color development in 3,3′-diaminobenzidine tetrahydrochloride (Dako, Carpinteria, CA). Finally, the slides were lightly counterstained with hematoxylin, dehydrated with ethanol, cleaned with xylene, and mounted. To avoid non-specific binding, an immunizing peptide blocking experiment was performed. As a negative control, triplicate sections were immunostained without exposure to primary antibodies, thus confirming the staining specificity [42]. To quantify the level of FOXA1 protein expression in those stained slides, we used a previously described IHC scoring system with minor modification [43], [44]. Briefly, the mean percentage of FOXA1-positive tumor cells in each section was determined by analysis of at least five random fields at ×400 magnification. The intensity of the FOXA1 immunoreactive signal was scored as follows: 1+, weak; 2+, moderate; 3+, intense. The percentage of positive tumor cells and the staining intensity of each section were then multiplied to produce the FOXA1 IHC score. Cases with a FOXA1 IHC score >123.0 (the highest score for normal tissue) were defined as positive. These judgments were made by two independent pathologists, neither of whom had any knowledge or information pertaining to the patients’ clinical status.

### Growth Assay

Cells were trypsinized and equal numbers (1×10^4^ cells) were seeded in a 24-well culture plate for all transfections. After 24 h, the media was changed to fresh complete media (10% FBS) and cells were transfected with siRNA using Lipofectamine™ RNAiMax reagent (Invitrogen) or FOXA1 expressing vector (Thermo Scientific Cat #MHS1010-7296058 CO USA) using Lipofectamine™2000, according to the manufacturer’s instructions and incubated for 4 days. The cells were trypsinized and counted at each indicated time point. To study the effect of bicalutamide treatment, LNCaP or C4-2 cells were seeded and transfected with siRNA. After 48 h, the media was changed to the indicated concentrations of bicalutamide for 24 h. After incubation, the cells were trypsinized and counted.

### Enzyme-linked Immunosorbent Assays (ELISAs)

LNCaP or C4-2 cells were seeded and transfected with siRNA. After 72 h, concentration of IGFBP-3 in the media was measured using Human IGFBP-3 Quantikine ELISA Kit (R & D Systems), according to the manufacturer’s instructions.

### Cell Cycle Analysis

Cells were fixed with 70% ethanol (30 min at 4°C), stained with propidium iodide (50 µg/mL) containing 20 µg/mL of RNase (30 min at room temperature), and were subsequently analyzed by flow cytometric analysis using a FACS Canto™ II (Becton Dickinson). The fractions of the cells in the G0–G1, S, and G2–M phases were analyzed using Flow Jo software (Tree Star, Ashland, OR).

### Target Gene Search for FOXA1

The human 44 K oligo-microarray (Agilent Technologies, Santa Clara, CA, USA) was used for expression profiling of siFOXA1-transfected cells and of nega-transfected cells. Hybridization and wash steps were performed according to Agilent protocols. The arrays were scanned using a Packard GSI Lumonics ScanArray 4000 (Perkin Elmer, Boston, MA, USA). The microarray data were analyzed using DNASIS array software (Hitachi Software Engineering, Tokyo, Japan), which converted the signal intensity of each spot into text files. The log2 ratios of the median subtracted background intensities were analyzed. Functional categories to which the up-regulated genes belonged were analyzed using gene-analysis software DAVID (http://david.abcc.ncifcrf.gov/). The DAVID functional annotation tool [45], [46] was used to classify gene sets based on Gene Ontology identifiers. Functional categories with a Benjamini-Hochberg statistic less than 0.05 were considered to be statistically significant.

### Statistical Analysis

Statistical significance of differences between groups was evaluated using the Mann-Whitney U test. Expert StatView (version 4, SAS Institute Inc., Cary, NC, USA) analysis software was used and P<0.05 was considered statistically significant. The data are expressed as means ± standard error. PSA failure was defined as the time when the PSA value of a patient that had undergone prostatectomy was over 0.2 ng/ml. The survival time was defined as the time between when the patient underwent radical prostatectomy and when the first PSA value of over 0.2 ng/ml was detected, or the date of the last follow-up, whichever occurred first. Patients without elevation of PSA during follow-up were considered to have a good prognosis; patients with PSA failure during follow-up were considered to have a poor prognosis. Survival curves were obtained using the Kaplan-Meier method and differences in survival rates between FOXA1-positive and FOXA1-negative cases, and between AR-positive and AR-negative cases, were compared using a log-rank test.

## Supporting Information

Figure S1 (A, B) LNCaP or C4-2 cells were transfected with a negative control siRNA (nega) or with one of three different FOXA1 siRNAs (siFOXA1#1–3). After 48 h of transfection PSA mRNA expression was analyzed by qRT-PCR. PSA mRNA expression is expressed relative to GAPDH mRNA expression in the same cell. (C, D) LNCaP or C4-2 cells were transfected with FOXA1 plasmide, and were then reseeded and cell proliferation assayed over 4 days by counting the number of cells. (E, F) LNCaP or C4-2 cells were transfected with a negative control siRNA (nega) or with a FOXA1 siRNA (siFOXA1 #3). 4 h before protein extraction, the media was changed to IGF-1 contained media (100 ng/ml). After 72 h of transfection, proteins were extracted and the expression of phospho-Akt, total amount of Akt and GAPDH was examined by western blot analysis. These results are the means ± S.E.M. of three independent experiments. **indicates a statistical difference of P<0.01.
(TIF)
