# Supplementary figures and images for: FOXA1 Promotes Tumor Progression in Prostate Cancer via the Insulin-Like Growth Factor Binding Protein 3 Pathway
Source: PLoS One. 2012 Aug 3;7(8):e42456. doi: 10.1371/journal.pone.0042456 (PMC3411739; doi:10.1371/journal.pone.0042456)

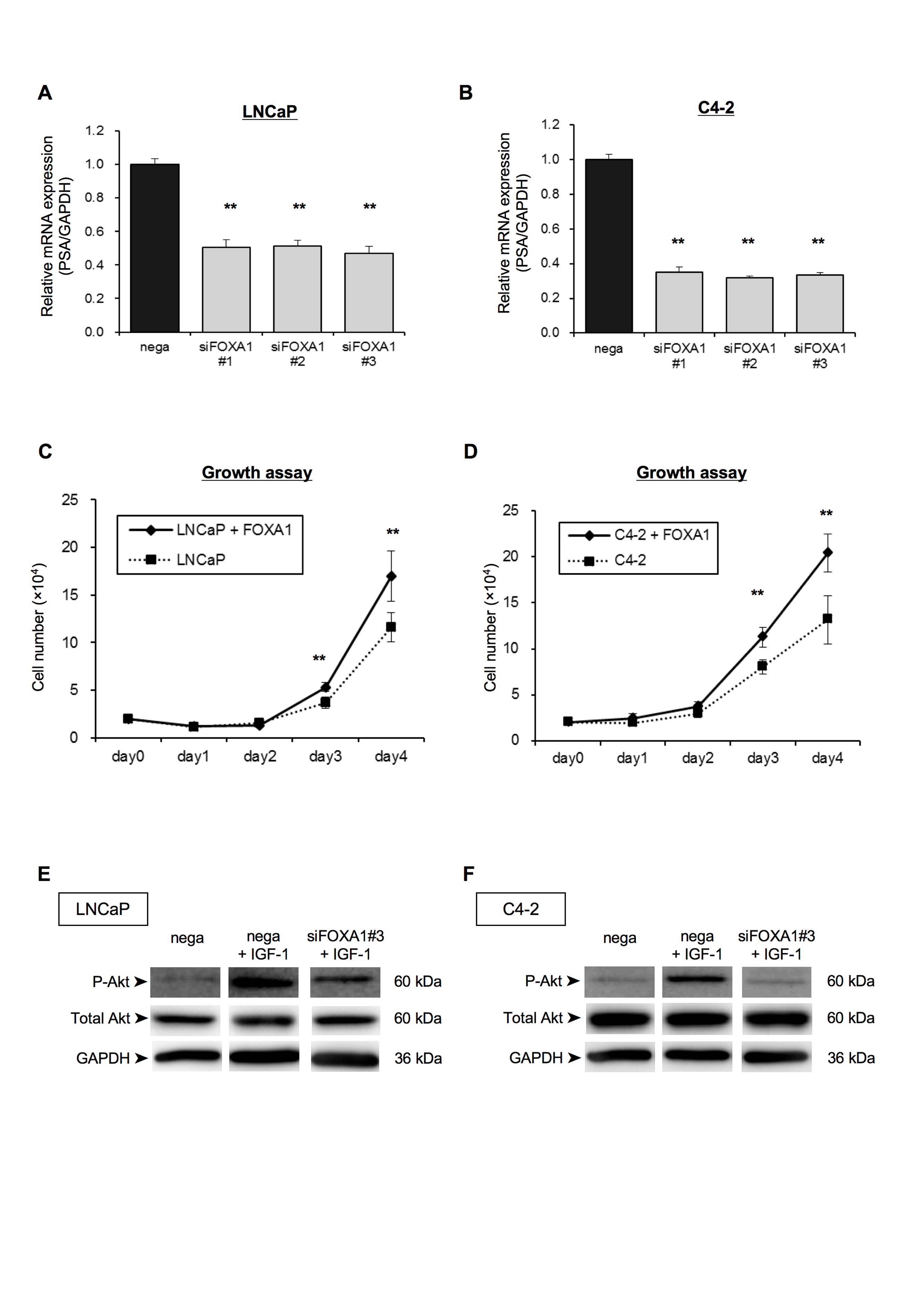

Supplement: Figure S1 — (A, B) LNCaP or C4-2 cells were transfected with a negative control siRNA (nega) or with one of three different FOXA1 siRNAs (siFOXA1#1–3). After 48 h of transfection PSA mRNA expression was analyzed by qRT-PCR. PSA mRNA expression is expressed relative to GAPDH mRNA expression in the same cell. (C, D) LNCaP or C4-2 cells were transfected with FOXA1 plasmide, and were then reseeded and cell proliferation assayed over 4 days by counting the number of cells. (E, F) LNCaP or C4-2 cells were transfected with a negative control siRNA (nega) or with a FOXA1 siRNA (siFOXA1 #3). 4 h before protein extraction, the media was changed to IGF-1 contained media (100 ng/ml). After 72 h of transfection, proteins were extracted and the expression of phospho-Akt, total amount of Akt and GAPDH was examined by western blot analysis. These results are the means ± S.E.M. of three independent experiments. **indicates a statistical difference of P<0.01. (TIF) [file pone.0042456.s001.tif]
